# Isolated abdominal wall metastasis 42 years after curative surgery for ovarian cancer: A case report

**DOI:** 10.1016/j.crwh.2025.e00696

**Published:** 2025-02-20

**Authors:** Roland Csorba, Paul Buderath, Marc Ingenwerth, Sa'ed Almasarweh, Zeynep Atas Elfrink

**Affiliations:** a Department of Gynecology and Obstetrics, Essen University Hospital, Essen, Germany; b Department of Pathology, Essen University Hospital, Essen, Germany

**Keywords:** Ovarian cancer, Abdominal wall metastasis, Late recurrence, Long-term follow-up

## Abstract

Despite the rarity of abdominal or chest wall metastases in ovarian cancer patients, reports have described instances of isolated late recurrence at surgical incision sites. We report the case of an 85-year-old woman who present with a massive metastatic tumor on the right anterior abdominal wall 42 years after undergoing a total abdominal hysterectomy and bilateral salpingo-oophorectomy for primary ovarian cancer. The abdominal wall tumor was resected en bloc, and abdominal wall reconstruction was performed using a mesh. Histology revealed a low-grade serous carcinoma.

This report highlights the possibility of abdominal wall metastases after prolonged survival following the treatment of ovarian cancer. Surgical excision combined with mesh reconstruction represents an adequate treatment approach for such cases. Caution should be exercised during laparotomy to ensure complete removal of malignant tissue and to prevent parietal dissemination. Long-term follow-up is crucial for ovarian cancer patients, as late recurrences, although rare, can occur even decades after initial treatment.

## Highlights

• The report highlights the possibility of delayed abdominal wall metastases following the treatment of ovarian cancer.
• Surgical excision combined with mesh reconstruction represents an adequate treatment approach for such cases.
• Caution should be exercised during laparotomy to R0-Resection and to prevent parietal dissemination.
• Long-term follow-up is crucial for ovarian cancer patients, as late recurrences can occur after initial treatment.

## 1 Introduction

Ovarian cancer is the eighth most commonly diagnosed cancer and one of the leading causes of death in women with gynecological malignancies [1]. Most patients present in late stages and prognosis is generally poor, with a relative 5-year survival of 44 % [2]. Despite the rarity of abdominal or chest wall metastases in ovarian cancer patients, reports have described instances of isolated late recurrence at surgical incision sites [3,4]. However, the appearance of abdominal wall metastases (AWM) more than 30 years after optimal surgical cytoreduction, which includes, but is not limited to, total abdominal hysterectomy, bilateral salpingo-oophorectomy, omentectomy, peritonectomy and, in selected cases, pelvic and para-aortic lymphadenectomy, is exceptionally rare.

In this report, we present an unusual case of an isolated abdominal wall low-grade serous ovarian cancer metastasis in an ovarian cancer survivor who had undergone curative surgery more than 40 years previously. The case highlights the importance of long-term follow-up and the potential for late recurrences in ovarian cancer patients, even after extensive surgical intervention and a prolonged disease-free interval.

## 2 Case Presentation

An 85-year-old woman presented with a 5-month history of chronic pain and swelling in the middle right abdomen, without associated gastrointestinal symptoms. The abdominal wall mass was first noticed 6 months prior.

**Fig. 1:**
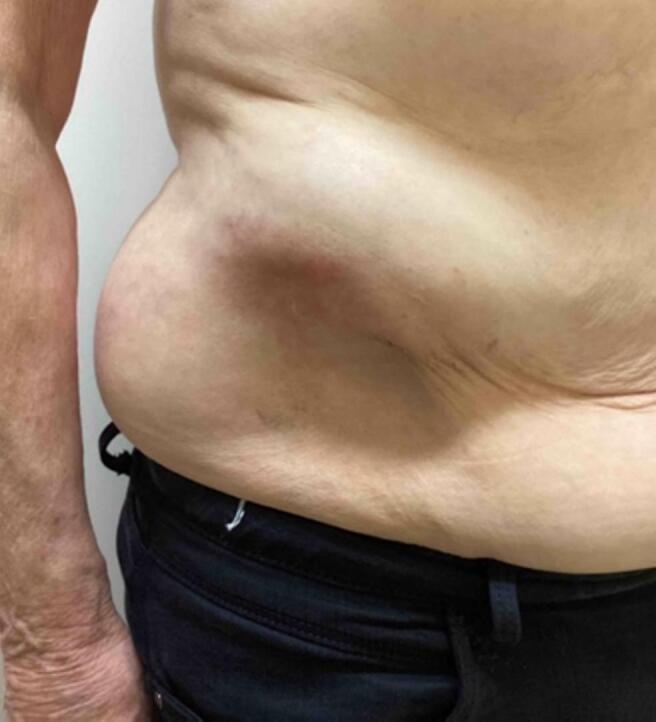
A non-tender, 10-cm soft-tissue palpable mass in the lower right rectus abdominis muscle.

At the age of 43 years, the patient underwent total cytoreductive surgery via laparotomy for FIGO stage Ia ovarian cancer, which included a total abdominal hysterectomy, bilateral salpingo-oophorectomy, omentectomy, and pelvic and para-aortic lymphadenectomy. The tumor was not graded at that time, and no adjuvant chemotherapy was administered. The original histologic report could not be retrieved. The patient's medical history was otherwise unremarkable, with no reported comorbidities or allergies. Physical examination revealed a non-tender, 10-cm soft-tissue mass palpable in the lower right rectus abdominis muscle (Fig. 1).

**Fig. 2:**
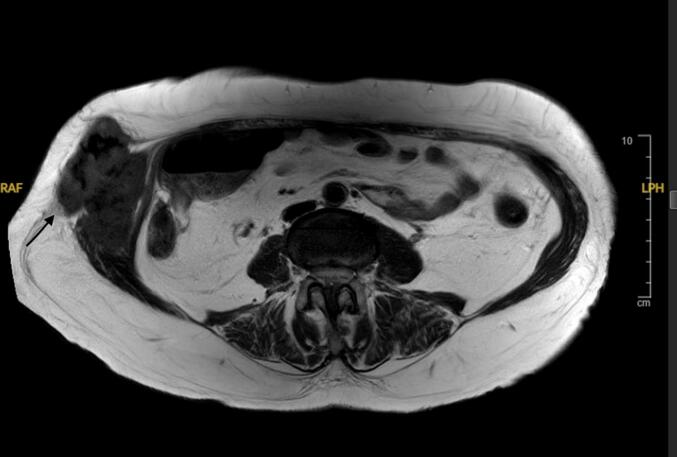
Preoperative T1-weighted MRI revealed a heterogeneous tumor slightly growing in the right rectus abdominis (black arrow).

Transabdominal ultrasonography confirmed the location and showed a well demarcated solid mass. Magnetic resonance imaging (MRI) revealed a heterogeneous tumor slightly growing in the right rectus abdominis (Fig. 2).

Laboratory tests on admission showed a reduced hematocrit of 26 % and a serum hemoglobin concentration of 92 g/L, with a normal platelet count.

**Fig. 3:**
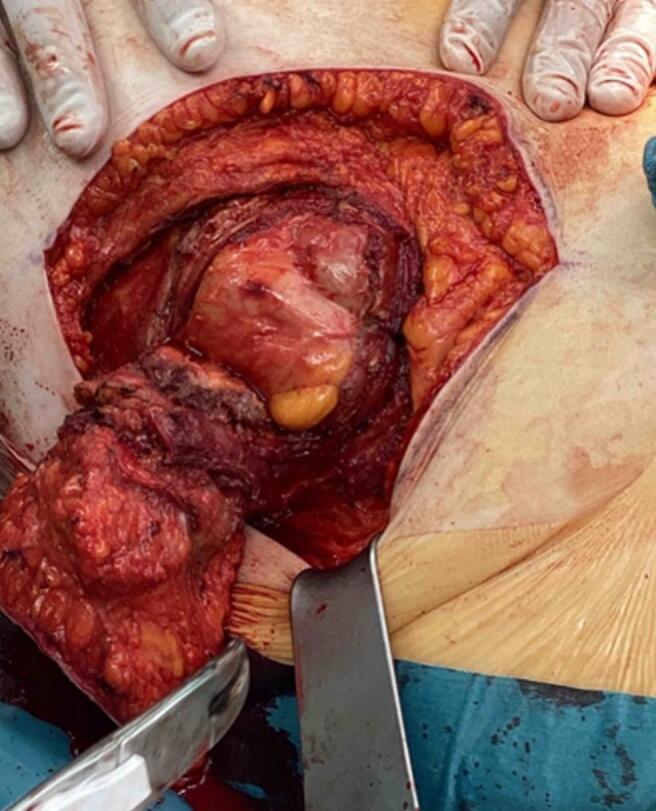
Intraoperative photograph of the abdominal wall tumor excised.

**Fig. 4:**
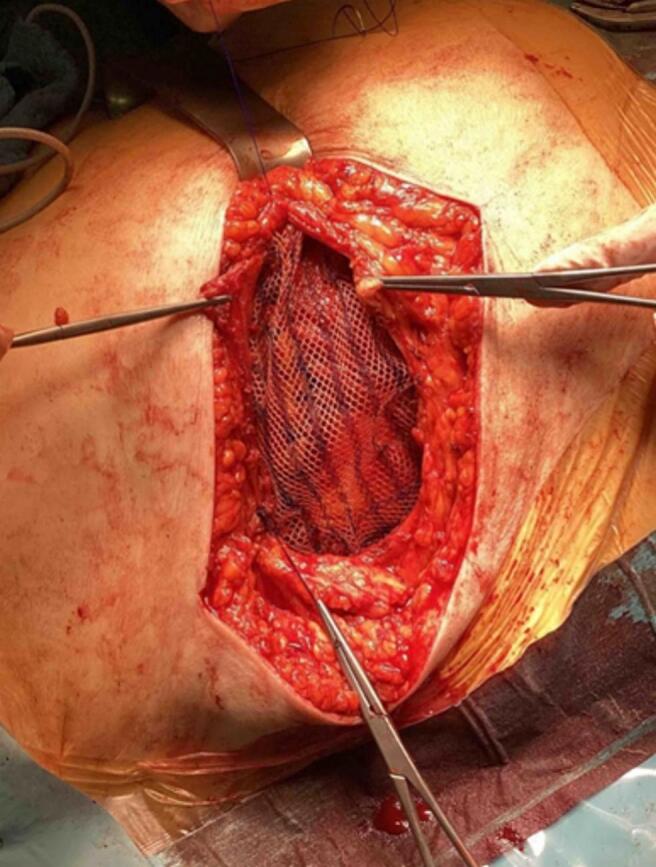
Intraoperative use of a synthetic mesh covering the defect in the rectus sheath.

After obtaining patient consent, an elective laparotomy was performed under general anesthesia. The abdominal wall tumor was excised with wide margins, and the defect in the rectus sheath was repaired using a synthetic mesh (Fig. 3, Fig. 4).

**Fig. 5:**
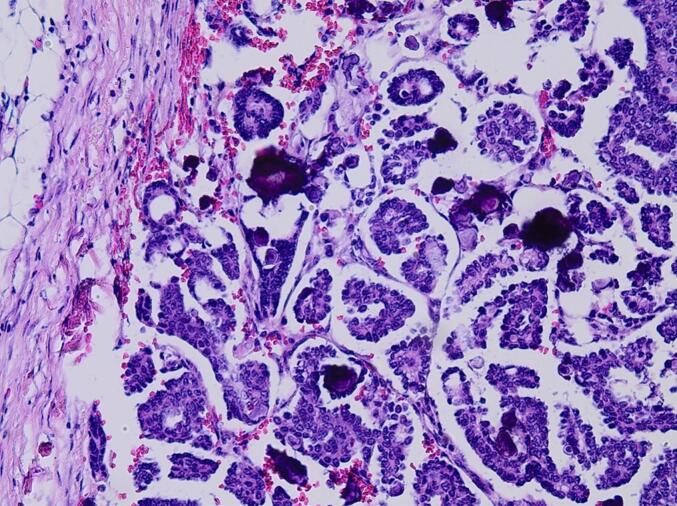
Representative microphotograph of the low-grade serous carcinoma. The tumor is composed of thin papillae and nuclei show mild to moderate nuclear atypia. Frequent psammoma bodies can be seen. No necrosis. (Hematoxylin and eosin stain, 20×).

Pathological examination of the excised tumor confirmed the presence of metastatic low-grade serous ovarian carcinoma, with clear surgical margins free of malignant cells (Fig. 5). Diagnosis was confirmed by immunistochemistry with positive staining for CK7, PAX8, estrogen receptor and wild-type staining pattern for P53. Negative stains included TTF1, thyreoglobulin, napsin A and calretinin.

The patient's postoperative course was uneventful and prophylactic antibiotic treatment was deemed unnecessary. She demonstrated a satisfactory recovery and was discharged from the hospital on the third day following the surgical intervention.

Postoperatively, the patient was referred for a discussion of adjuvant treatment options, given her FIGO stage IV diagnosis. During the consultation, chemotherapy was presented as a potential option to reduce the risk of recurrences. After considering the risks and benefits of all available treatment options, the patient expressed high satisfaction with her postoperative outcomes and emphasized her desire to prioritize her quality of life, particularly at the age of 85. In light of these considerations, the shared decision was made to forgo adjuvant treatment, including chemotherapy.

## 3 Discussion

Isolated abdominal wall metastases following ovarian cancer surgery are considered rare. The incidence of parietal metastasis after ovarian cancer surgery is increased by laparoscopic approaches [3]. Port site metastases are a well-documented and significant complication after laparoscopic resection of intra-abdominal malignancies. The incidence of abdominal wall recurrence in patients after laparotomy is unknown but is considered rare.

The advantages of minimally invasive surgery, including less pain, quicker recovery, and shorter hospitalization time, have been realized in patients with intra-abdominal malignancies. However, concerns about port-site metastases have limited the application of laparoscopic techniques to the resection of intra-abdominal malignancies. Port site metastases represent significant patient morbidity. The first report of port site metastatic disease was in 1978 in a patient who underwent diagnostic laparoscopy for ovarian cancer [5]. In ovarian cancer, the incidence of port site metastases after laparoscopic surgery is reported to range from 16 % to 47 % [6].

In the follow-up for ovarian cancer, it is essential to regularly monitor the patient's condition through physical examinations, imaging studies, and tumor marker assessments. The present patient did not adhere to the recommended follow-up schedule, which led to a delayed detection of the lesion. Management depends on the patient's condition, the size of the metastasis, previous operations, comorbidities, and the expertise of the surgeon. Conventionally, the best treatment for localized metastasis is surgical resection, supported by chemotherapy [[7], [8], [9]]. The challenges associated with AWM resection include the complete removal of the lesion, ensuring rapid wound healing and maintaining the integrity of the abdominal wall. For cases similar to the one presented here, a laparotomy with wide excision is strongly recommended. Synthetic mesh can be used for surgical repair of the abdominal wall defect.

## 4 Conclusion

Extreme caution is recommended when removing a malignant neoplasm from the abdomen during open surgery or laparoscopy. The difficulty lies in avoiding any peritoneal and parietal dissemination while extracting the tumor. Good surgical technique, not only during laparoscopic operations but also during open surgery, can reduce the risk of abdominal wall metastasis.
